# Primer development to obtain complete coding sequence of HA and NA genes of influenza A/H3N2 virus

**DOI:** 10.1186/s13104-016-2235-8

**Published:** 2016-08-30

**Authors:** Agustiningsih Agustiningsih, Hidayat Trimarsanto, Vivi Setiawaty, I. Made Artika, David Handojo Muljono

**Affiliations:** 1 National Institute of Health Research and Development, Ministry of Health Republic of Indonesia, Jl. Percetakan Negara No.23, Jakarta, 10560 Indonesia; 2 Eijkman Institute for Molecular Biology, Jl. Diponegoro No.69, Jakarta, 10430 Indonesia; 3 Agency for the Assessment and Application of Technology, Jl. M. H. Thamrin 8, Jakarta, 10340 Indonesia; 4 Department of Biochemistry, Faculty of Mathematics and Natural Sciences, Bogor Agricultural University, Darmaga Campus, Bogor, West Java 16680 Indonesia; 5 Faculty of Medicine, Hasanuddin University, Jl. Perintis Kemerdekaan Km 10, Makassar, South Sulawesi 90245 Indonesia

**Keywords:** HA gene, Influenza A/H3N2 virus, NA gene, Primer set

## Abstract

**Background:**

Influenza is an acute respiratory illness and has become a serious public health problem worldwide. The need to study the HA and NA genes in influenza A virus is essential since these genes frequently undergo mutations. This study describes the development of primer sets for RT-PCR to obtain complete coding sequence of Hemagglutinin (HA) and Neuraminidase (NA) genes of influenza A/H3N2 virus from Indonesia. The primers were developed based on influenza A/H3N2 sequence worldwide from Global Initiative on Sharing All Influenza Data (GISAID) and further tested using Indonesian influenza A/H3N2 archived samples of influenza-like illness (ILI) surveillance from 2008 to 2009.

**Results:**

An optimum RT-PCR condition was acquired for all HA and NA fragments designed to cover complete coding sequence of HA and NA genes. A total of 71 samples were successfully sequenced for complete coding sequence both of HA and NA genes out of 145 samples of influenza A/H3N2 tested.

**Conclusions:**

The developed primer sets were suitable for obtaining complete coding sequences of HA and NA genes of Indonesian samples from 2008 to 2009.

**Electronic supplementary material:**

The online version of this article (doi:10.1186/s13104-016-2235-8) contains supplementary material, which is available to authorized users.

## Background

Influenza is a major cause of mortality and morbidity throughout the world. Clinical symptoms of influenza infection are often similar to the manifestation of other respiratory viral infections, and therefore influenza virus infection cannot be reliably diagnosed from its clinical symptoms [1]. The early detection of influenza virus in the early stage of infection is important as treatment with antiviral needs to be initiated soon after the onset of disease [2].

Influenza virus is usually detected by isolation of the virus from respiratory specimen in cell culture, commonly Madin–Darby canine kidney (MDCK) cell line, and the presence of virus is confirmed by hemagglutination test [3]. Although virus culture is sensitive, it requires viable virus and considerable time to perform [1]. Other molecular methods for the diagnosis of influenza virus have been developed, of which methods based on polymerase chain reaction (PCR) have the highest specificity and sensitivity for viral genome detection [4].

Reverse transcriptase PCR (RT-PCR) assay has been used to detect negative sense RNA viruses including influenza viruses. RT-PCR has been applied to identify the type and subtype of influenza virus cultivated in cell culture or directly in clinical samples. A PCR assay can be performed within approximately 2–3 h, a much shorter time compared to viral culture which requires approximately 2–3 days. The assay specificity can be increased by designing sequence-specific primers based on the region of interest within the viral genome [5].

Sequence analysis is specifically needed in relation to evolutionary pattern analysis. RT-PCR enables to carry out this analysis as the PCR product can be directly sequenced to obtain the genetic sequences from a particular region. Hemagglutinin (HA) and neuraminidase (NA) are the essential objects of analysis in this study, since they are the most frequent genes that undergo mutations [6]. Consequently, particular attention should be given to the primer sets used to amplify these genes, because the high nucleotide variability in HA and NA genes may affect the primer binding during PCR [4].

This study describes the development of PCR assay method to amplify and obtain complete coding sequence of HA and NA genes of Influenza A/H3N2 virus from Indonesia in 2008 to 2009.

## Methods

### Sample selection

The study samples were 145 archival clinical specimens confirmed for H3N2 that were obtained from the influenza-like illness (ILI) surveillance conducted by the National Institute of Health Research and Development (NIHRD), Indonesia, from 2008 to 2009. Influenza virus subtyping for H3N2 confirmation was performed previously using real-time RT-PCR according to WHO protocol for influenza surveillance. All samples were suspended in hank balance salt solution (HBSS) transport medium and were stored properly in −80 °C until used.

### Primer design and optimization

**Fig. 1:**
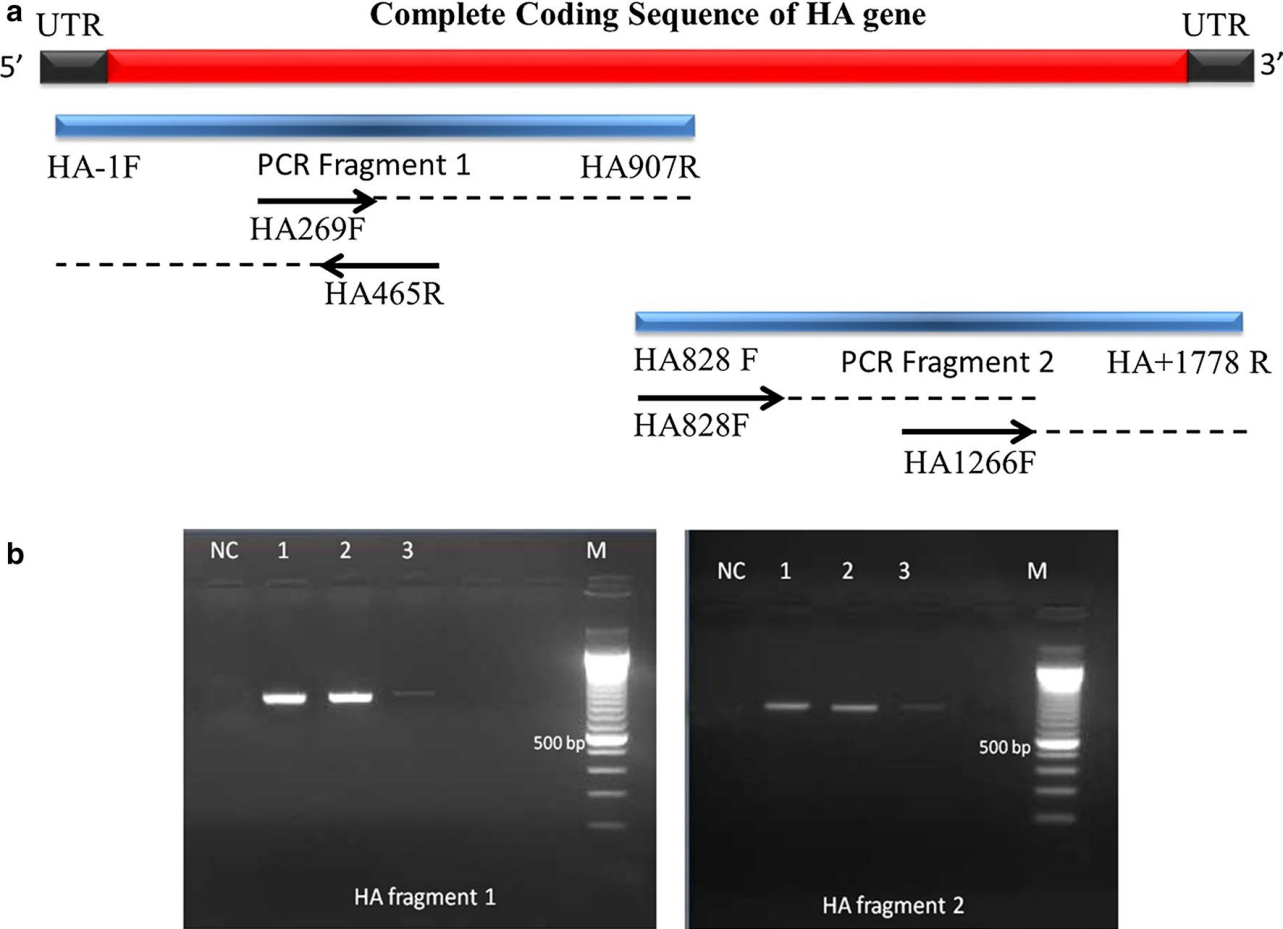
HA RT-PCR product. **a** Schematically, the designed primer sets generate overlapping fragments (*blue bars*) to obtain the full length of HA gene (*red bars*). *Black bars* represent the untranslated region (UTR). The *black arrows* represent the inner primers for sequencing to obtain complete coding sequences. **b** The HA1 and HA2 fragments were approximately 900 bp in length. NC represents negative control. *Line 1*, *2*, and *3* represent the annealing temperatures of 50, 55 and 60 °C, respectively. M represents DNA Marker with size increment of 100 bp

**Fig. 2:**
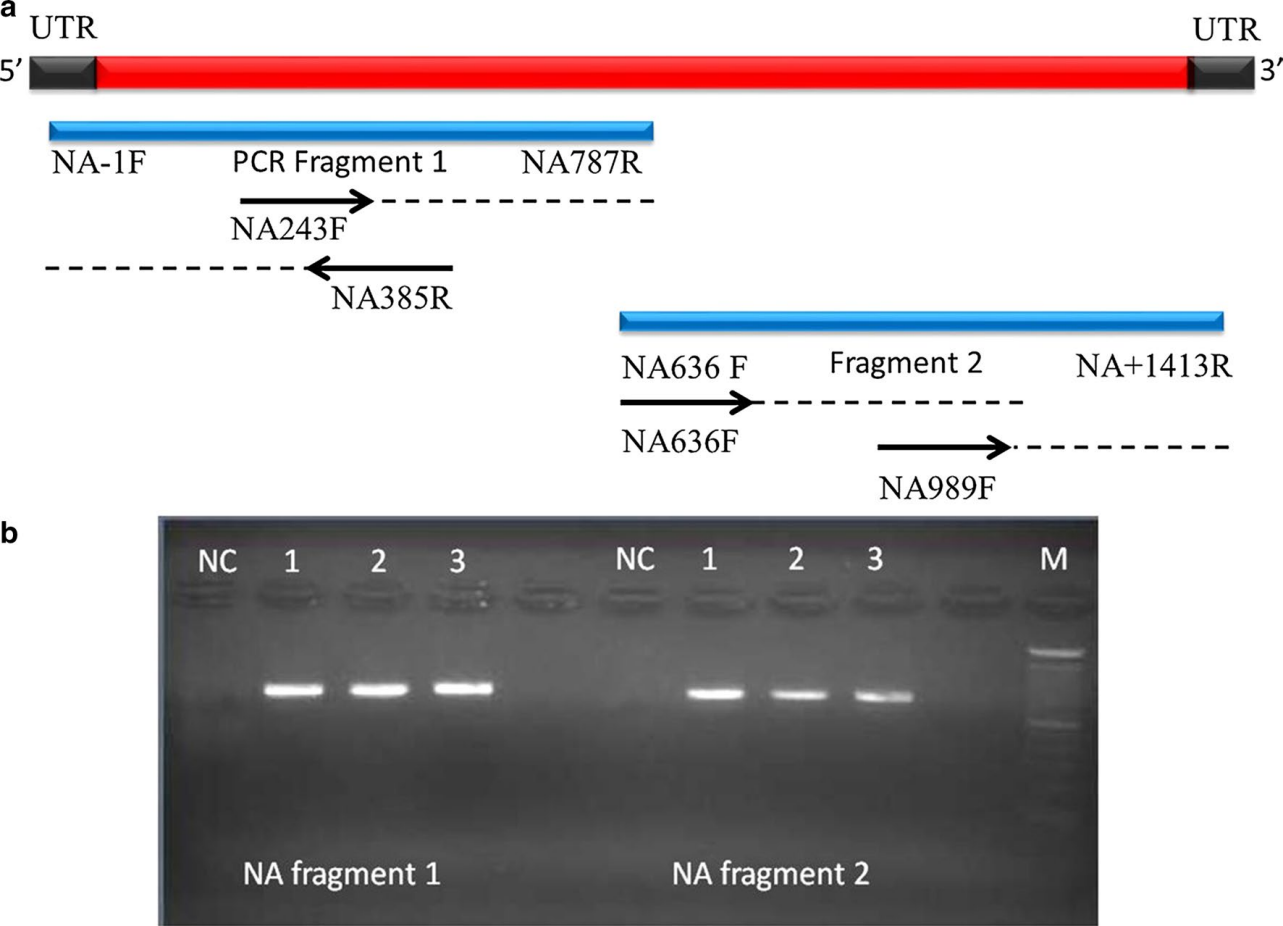
NA RT-PCR product. **a** Schematically, the designed primer sets generate overlapping fragments (*blue bars*) to obtain the full length of NA gene (*red bars*). *Black bars* represent the untranslated region (UTR). The *black arrows* represent the inner primers for sequencing to obtain complete coding sequences. **b** The NA1 and NA2 fragments were approximately 700 bp in length. NC represents negative control. *Line 1*, *2*, and *3* represent the annealing temperatures of 50, 55 and 60 °C, respectively. M represents DNA Marker with size increment of 100 bp

Primer sets used to detect H3N2 virus in this study were developed based on the complete coding sequence of HA and NA of influenza A genes [7]. Approximately 2000 H3N2 sequences from five continents from 1982 to 2009 were retrieved from Global Initiative on Sharing All Influenza Data (GISAID) database to aid the design of the primer sets to ensure maximum detection. The list of GISAID sequences used in this report is available in Additional file 1. Primer dimers and other secondary structures of the designed primer were checked using PerlPrimer v1.1.18 [8]. BLAST was used to compare the primer sequence with the expected target sequence in GenBank. To increase the rate of successful amplification, both complete coding sequences of HA and NA genes were amplified into two shorter fragments, resulting in approximately 900 bp of HA fragments (HA1 and HA2 fragments) and 700 bp of NA gene fragments (NA1 and NA2 fragments) (Figs. 1 and 2). These two fragments were designed to have overlapping regions to assist in assembling the fragments into complete coding sequences of the respective genes.

PerlPrimer v1.1.18 was employed to calculate the melting temperature (Tm) of each primer, which then was used to define the initial annealing temperature for optimizing the RT-PCR condition and obtaining optimum PCR products. The optimum annealing temperature of the primers sets for RT-PCR is approximately 5 °C higher than the Tm given by PerlPrimer v1.1.18 calculation. Three annealing temperatures with 5 °C differences were tested to obtain optimum PCR product, using H3N2 virus from positive samples isolated in MDCK cell line. The optimization of the primer sets were also performed by running the parallel positive samples in gradient cycler on gradient temperature from 48 to 60 °C.

### RNA isolation and RT-PCR

Purification of viral RNA was carried out using TRIZOL™ (Invitrogen, Carlsbad, CA) according to the manufacturer’s instruction, and suspended in 50 µL of RNase-free water. RT-PCR was conducted using primers shown in Table 1 using SuperScript™ III One-Step RT-PCR System with Platinum *Taq* DNA Polymerase (Invitrogen, Carlsbad, CA). RT-PCR was performed on C1000 PCR Thermal cycler (Bio-Rad, Hercules, CA) in a total reaction volume of 25 μL. The reaction contains of 1× PCR buffer, 200 μM dNTPs, 1.6 mM MgSO4, 0.2 μM of each forward and reverse primer, 1 μL SuperScript™ III RT and Platinum^®^*Taq* mix and 5 μL of purified RNA. RT-PCR for ILI samples were performed under the following conditions: reverse transcription at 55 °C for 30 min, hot start at 94 °C for 2 min and 40 cycles of PCR consisting of denaturation at 94 °C for 30 s, primer annealing at 50 °C for 30 s and extension at 72 °C for 1 min and ended with a final extension at 72 °C for 5 min. The expected PCR products were analyzed by electrophoresis using 1 % of agarose stained with ethidium bromide.

**Table 1:** List of primers used for sequencing

Fragment	Primer	Sequence (5′-3′)	Location	Tm (°C)
HA1	HA-1F^a^	CTCGAGAGCAAAAGCAGGGG	5′ end	65.62
	HA269F	CTCAGTGTGATGGCTTCCAA	269–288	
	HA465 R	GTTATTAGATCTCCTTATG	465–483	
	HA907R^a^	GGTTTGTCATTGGGAATGCT	907–926	61.22
HA2	HA828F^a^	ACGAAGTGGGAAAAGCTCAATA	828–849	62.31
	HA1266F	TCAGGACCTTGAGAAATATGTTG	1266–1288	
	HA + 1778R^a^	AGTAGAAACAAGGGTGTTTT	3′ end	57.15
NA1	NA-1F^a^	GAGCAAAAGCAGGAGTAAAG	5′ end	59.13
	NA243F	AGCAGAATACAGAAATTGGTC	243–263	
	NA385R	GTCAGGATCGCATGACACAT	385–406	
	NA787R^a^	TGACAATGTGCTAGTATGAAC	787–807	58.05
NA2	NA636F^a^	AGATAGTGTTGTTTCATGGTC	636–656	57.96
	NA989F	ACAGCTCCAGCAGTAGCCATTG	989–1010	
	NA + 1413R^a^	AGTAGAAACAAGGAGTTTTT	3′ end	54.63

^a^ Primers used for RT-PCR

### DNA sequencing of HA and NA genes

Direct sequencing was performed using PCR products that were purified using QIAquick™ PCR purification kit (QIAGEN, Hilden Germany) according to the manufacturer’s instruction. DNA sequencing was carried out using the Big Dye Terminator V.3.0 Cycle Sequencing Ready Reaction Kit (Applied Biosystem, Foster City, CA) together with the primers listed in Table 1 for each PCR fragments on ABI 3130xl Genetic Analyzer automatic sequencer (Applied Biosystems, Foster City, CA, USA). The nucleotide sequences were edited, assembled and aligned using BioEdit Sequence Alignment Editor Ver 7.0.5.2 [9].

## Results

Optimization for the developed primer sets to amplify the HA and NA fragments was performed in three different annealing temperatures: 50, 55 and 60 °C. These various temperatures were selected based on the Tm of the developed primers for RT-PCR listed in Table 1. The result of optimization using gradient cycler listed in Additional file 2. As shown in Figs. 1 and 2, the annealing temperatures 50 and 55 °C showed optimum results for all of HA and NA fragments in comparison with the annealing temperature 60 °C. In addition, both of HA and NA fragments did not show any unspecific bands and primer dimers, suggesting that the developed primers were specific for amplifying HA and NA genes of H3N2 virus. Therefore, the annealing temperature 50 °C was selected for further PCR testing of HA and NA genes for Indonesian samples.

Figures 1 and 2 illustrated the schematic arrangements of inner primer locations for DNA sequencing. Each PCR product covering partial length of either HA and NA genes was subjected directly to sequencing and by assembling the DNA sequences of the PCR product, the complete coding sequences of HA and NA genes were obtained.

A total of 145 of archival H3N2-confirmed samples from 2008 to 2009 were listed and then grouped according to geographical origin (Table 2). Unfortunately, not all of the archived clinical samples of ILI surveillance from 2008 to 2009 were successfully amplified and sequenced as listed in Table 2. Overall, complete coding sequencing was successfully performed on 45.9 % (51) and 58.8 % (20) of samples from 2008 to 2009, respectively.

**Table 2:** Sample distribution based on geographical origins

Year	Sumatra	Java	Kalimantan	Timor	Celebes	Papua	Total
2008	15 (8)	35 (17)	6 (5)	–	34 (12)	21 (9)	111 (51)
2009	10 (5)	4 (3)	8 (6)	4 (2)	7 (3)	1 (1)	34 (20)
Total	25 (13)	39 (20)	14 (11)	4 (2)	41 (15)	22 (20)	145 (71)

Number in bracket represents the number of the sequenced samples

The proportion of samples successfully sequenced varied across island groups, with approximately 50 % of samples from Sumatra (13), Java (20) and Timor (2), 78 % (11) from Kalimantan, 36.5 % (15) from Celebes and 45.5 % (10) from Papua.

## Discussion

Diagnostic methods for influenza infection that are routinely performed such as virus isolation and antigen detection are both sensitive and specific. The presence of molecular techniques for detection of influenza virus provides advantages for the investigation of respiratory outbreaks and may be essential for further epidemiology purposes such as evolutionary studies. This is the first report describing the development of primer sets to obtain complete coding sequence of HA and NA genes of influenza A/H3N2 virus that used Indonesian virus. As an archipelago country, the development of primer sets covering complete coding sequence of influenza A/H3N2 virus for further evolutionary studies has become a challenge in Indonesia. Maintaining the cold chain stability during specimen shipment become crucial for ILI surveillance in Indonesia.

The designed primer sets covering complete coding sequence of HA and NA gene of influenza A/H3N2 virus were developed based on the consideration that shorter PCR fragment gives effective PCR reaction compared with long fragment amplification. The most efficient amplification is in the 300–1000 base pair length. Given that not all of the samples of ILI surveillance were in good quality, efficient amplification of less than 1000 base pair fragment is become an important consideration in developing the primer sets to obtain complete coding sequence of HA and NA gene of H3N2 virus in Indonesia.

PCR has been recognized to have higher sensitivity and specificity compared to virus isolation and antigenic detection. Positive results of influenza virus in experimentally infected turkey was shown from day 3 to 10 post-challenge by virus isolation and from day 7 to 10 post-challenge by antigen detection using antigen capture enzyme immunoassay. In comparison, successful detection of influenza virus in the experimentally infected turkey using RT-PCR was made from day 3 to day 12 post-challenge, suggesting the higher sensitivity of RT-PCR compared to virus isolation and antigen detection [10].

Prior to sample examination, the RT-PCR primer sets used in this study were constructed based on the templates used for PCR amplification of the complete coding sequence of HA and NA genes [7]. This primer sets were re-designed to generate two overlapping PCR products (Figs. 1 and 2). To prevent any primer mismatches, sequences located in conserved region within HA and NA genes were selected for primer binding locations as listed in Table 1. The design of these primer pairs for RT-PCR was based on simple rules for efficient primers that the primer pairs were only 18-20 nucleotide-long with 50–60 % G + C composition. Moreover, the primer pairs were also designed to prevent any complementary sequence at the 3′ ends between primer pairs that may promote the formation of primer-dimer artifacts and reduce the yields of the desired product [11, 12].

Possible reason of only 45.9 and 58.8 % successful sequenced samples in this study could be due to the low virus concentration in the samples. It is also possible that the virus titer had continued to decrease throughout the year during the samples storage as repeated freezing and thawing might reduce the possibility to recover the virus. The low yield of PCR product could also be due to the degradation of the viral RNA. The poor condition of cold-chain during specimen handling and shipment from local surveillance teams in many remote areas to laboratory in Jakarta was one of the possible reasons that might lower the samples quality.

To ensure the negative result in this study, we have performed additional RT-PCR using primer sets developed previously [7] to the 5 clinical samples that showed negative results using designed primers sets. The result including the real-time RT-PCR CT value (obtained from ILI surveillance result, not done in this study) was listed as Additional file 3. The conservation of the primer binding site is one of the issues that could cause primer mismatches and negative results. However, the designed primer sets aligned with HA and NA genes of H3N2 virus from 2005 to 2015 obtained from GISAID was conserved and illustrated in Additional files 4 and 5, respectively.

The annealing temperatures of 50  and 55 °C have been shown to generate optimum PCR products for HA and NA genes, respectively. Given that some samples were not amplified, annealing temperature of 50 °C was selected as the optimum annealing temperature. This was based on the consideration that lower temperature can increase primer binding to the template, although it may also increase the unintended extension of nucleotides at the 3′ end of the primers [11]. Moreover, inner primers within PCR fragments were also employed to sequence the overlapping RT-PCR products in an attempt to obtain the intended length of sequences of each gene.

The limitation of this study is that the primer sets were validated using samples collected only in 2008–2009. However the alignment of the influenza A/H3N2 virus from 2010 until 2015 obtained from GISAID and the primer binding site both for HA and NA genes designed in this study showed that the primer binding site were still conserved (Additional files 4 and 5).

## Conclusions

The developed primer sets can be used to amplify the HA and NA genes of Indonesian H3N2 isolates. This step is useful for further research of this virus, including sequencing and molecular analysis of HA and NA genes that have high nucleotide variability.

## Additional files

10.1186/s13104-016-2235-8 The list of HA and NA sequences retrieved from GISAID database. This supplementary file was GISAD acknowledge table consisted of the list of HA and NA sequences including the submitting laboratories.
10.1186/s13104-016-2235-8 Result of the primer sets optimization using gradient temperature. The table described the optimization experiment of samples in gradient cycler on gradient temperature from 48 to 60 °C.
10.1186/s13104-016-2235-8 Result of additional RT-PCR experiment on positive clinical samples using different primer sets. The table described the results of additional RT-PCR experiment using previously developed primer sets to verify that no amplicon could be obtained using designed primer sets from some of positive samples.
10.1186/s13104-016-2235-8 The alignment of designed primer sets with HA sequences of H3N2 virus retrieved from GISAID.
10.1186/s13104-016-2235-8 The alignment of designed primer sets with NA sequences of H3N2 virus retrieved from GISAID.
