# Supplementary material for: Primer development to obtain complete coding sequence of HA and NA genes of influenza A/H3N2 virus
Source: BMC Res Notes. 2016 Aug 30;9(1):423. doi: 10.1186/s13104-016-2235-8 (PMC5004302; doi:10.1186/s13104-016-2235-8)
Supplement: Supplementary file 2 — 10.1186/s13104-016-2235-8 Result of the primer sets optimization using gradient temperature. The table described the optimization experiment of samples in gradient cycler on gradient temperature from 48 to 60 °C. [file 13104_2016_2235_MOESM2_ESM.docx]

Supplement 2. Result of the primer sets optimization using gradient temperature

| Temperature  (°C)* | HA1 fragment | HA2 fragment | NA1 fragment | NA2 fragment |
| --- | --- | --- | --- | --- |
| 48 | positive | positive | unspecific band | unspecific band |
| 50.2 | positive | positive | positive | positive |
| 51 | positive | positive | positive | positive |
| 52.5 | positive | positive | positive | positive |
| 53.4 | positive | positive | positive | positive |
| 54.2 | positive | positive | positive | positive |
| 55.6 | positive | positive | positive | positive |
| 56 | positive | thin band | positive | positive |
| 58 | positive | thin band | positive | thin band |
| 59,3 | thin band | thin band | positive | thin band |
| 60 | thin band | thin band | positive | thin band |

*Each run for each fragment was performed in triplicates
