# Supplementary material for: Primer development to obtain complete coding sequence of HA and NA genes of influenza A/H3N2 virus
Source: BMC Res Notes. 2016 Aug 30;9(1):423. doi: 10.1186/s13104-016-2235-8 (PMC5004302; doi:10.1186/s13104-016-2235-8)
Supplement: Supplementary file 3 — 10.1186/s13104-016-2235-8 Result of additional RT-PCR experiment on positive clinical samples using different primer sets. The table described the results of additional RT-PCR experiment using previously developed primer sets to verify that no amplicon could be obtained using designed primer sets from some of positive samples. [file 13104_2016_2235_MOESM3_ESM.docx]

Supplement 3. Result of additional RT-PCR experiment on positive samples using different primer sets

| Sample No. | Real-time RT-PCR*  (CT value) | HA and NA designed primer sets | HA and NA primer sets from previous study (Hoffman, et al 2001) |
| --- | --- | --- | --- |
| DKI 066 | 27.34 | Negative | Negative |
| BM 062 | 33.41 | Negative | Negative |
| BM 712 | 32.05 | Negative | Negative |
| LB 597 | 27.68 | Negative | Negative |
| AC 323 | 30.56 | Negative | Negative |

*Positive real-time RT-PCR results were obtained from ILI surveillance data and not done in this study. Samples with CT value < 40 were positive for influenza A/H3N2 virus.
